# Translation of the Pictorial Fit-Frail Scale Into the Greek Language and Examination of Its Validity and Reliability

**DOI:** 10.7759/cureus.41553

**Published:** 2023-07-08

**Authors:** Panagiota Voukelatou, Andreas Kyvetos, Dafni Kollia, Pantelitsa Ellisaiou, Ioannis Vrettos

**Affiliations:** 1 2nd Department of Internal Medicine, General and Oncology Hospital of Kifissia “Agioi Anargyroi”, Athens, GRC

**Keywords:** reliability, validity, elderly, greek, pictorial fit-frail scale, frailty

## Abstract

Background: For the evaluation of frailty, a great variety of research tools are used internationally; however, only two have been translated and validated in Greek. The aim of the study was to translate the Pictorial Fit-Frail Scale (PFFS) into the Greek language and examine its validity and reliability.

Methods: Initially, the PFFS scale was translated into the Greek language through a six-step process. Subsequently, in a sample of 157 elderly patients (47.1% women), construct validity was examined with the known-groups method using the one-way ANOVA test and criterion concurrent validity by comparison with the Clinical Frailty Scale (CFS) using Pearson's correlation coefficient. Finally, inter-rater reliability and test-retest reliability were checked using the intraclass correlation coefficient.

Results: A comparison of known groups showed that older patients with greater dependence on activities of daily living, greater impairment of cognitive function, reduced mobility, balance, and swallowing disorders, as well as those who were socially withdrawn, scored higher on the PFFS scale, supporting the construct validity. The positive correlation between PFFS and CFS (r = 0.625, p ≤ 0.001) demonstrated the concurrent criterion validity of the PFFS scale. Intraclass correlation was excellent for both inter-rater reliability (0.951 (95% CI: 0.934-0.964)) and test-retest reliability (0.948 (95% CI: 0.930-0.962)).

Conclusion: The translated PFFS scale in Greek is a valid and reliable tool.

## Introduction

It has been stated that frailty, which is a clinical state in which there is an increase in an individual’s vulnerability for developing increased dependency and/or mortality when exposed to a stressor [1], is a well-recognized and common syndrome among older adults [1-3], and it may become one of the world's most serious health issues [4]. Not long ago, several European and US societies recommended all persons older than 70 years be screened for frailty [2]. Subsequently, the International Conference on Frailty and Sarcopenia Research (ICFSR) established evidence-based guidelines for identifying and managing physical frailty [3].

Although multiple reviews have highlighted the need for a standard tool for frailty assessment, several such tools are currently in use. The most widely used tools for the assessment of frailty are the gait speed test, the Clinical Frailty Scale (CFS), the physical frailty phenotype, the frailty index, and the Short Physical Performance Battery. The time to complete them varies from less than 10 minutes, when they are simple, to several hours, if the frailty assessment is done in stages and if special equipment is required. Moreover, many tools require patients to report their health issues or they measure physical performance, such as grip strength, in a manner that is inapplicable for older adults with communication problems [5-8]. Oviedo-Briones et al. [9], while comparing eight commonly used frailty assessment tools (i.e., Frailty Phenotype; Survey of Health, Ageing, and Retirement in Europe Frailty Instrument (SHARE-FI); three-item Frailty Trait Scale; five-item Frailty Trait Scale; FRAIL; 35-item Frailty Index; Gérontopôle Frailty Screening Tool; and CFS) in different clinical settings, have noted that the inter-scale agreement among them was only fair. Moreover, in another comparison of five frailty assessment tools (CFS, simple FRAIL questionnaire, PRISMA-7 (Program of Research to Integrate the Services for the Maintenance of Autonomy) questionnaire, Timed Up and Go Test, and Gérontopôle Frailty Screening Tool) with Fried phenotype being used as a reference standard, various levels of sensitivity, specificity, and accuracy for the diagnosis of frailty were demonstrated [10].

At this moment, there is no consensus regarding the best measure of frailty, and if the same tools could be used in different settings [11]. A reasonable approach for clinicians and researchers is the tool selection to be based on aspects of translation and validation for their country and suitability for their clinical setting [7]. Moreover, according to the ICFSR guidelines, both screening for frailty and also clinical assessment of frailty must be conducted using different tools [3]. Consequently, at least two different frailty instruments must be translated and validated in a language, intended for different purposes.

Recently, a simple-to-use and easy-to-administer frailty screening tool was developed. This is the Pictorial Fit-Frail Scale (PFFS), an image-based tool that has been designed to be sensitive to cultural differences [12]. The PFFS has comparable diagnostic accuracy [13,14] and proven validity and reliability in different clinical settings (community-dwelling older adults [13], hospitalized patients [14], and outpatients attending geriatric healthcare settings [15] or public primary healthcare clinics [16]) to other frailty assessment tools. Moreover, its unique characteristics (i.e., it does not require geriatric training and is applicable to people with limited health literacy or language barriers) could be helpful for frailty assessment in countries such as Greece, where the proportion of the population over 80 years is rising [17], the geriatricians are lacking, and the number of non-native speakers is high [18].

Even for a tool like PFFS, its translation is essential to avoid misclassifications due to cultural differences or individual perceptions of the English version [19], and indeed, PFFS has already been translated into Malaysian [16]. At this time, in Greece, only CFS [20] and Tilburg Frailty Indicator (TFI) [21] have been translated and validated, limiting the choices of clinicians and researchers. Due to the unique properties of PFFS, in an effort to promote its proper use for frailty assessment in Greece, we aimed to create a valid and reliable Greek version.

## Materials and methods

Obtaining the Greek version of the PFFS

**Figure 1:**
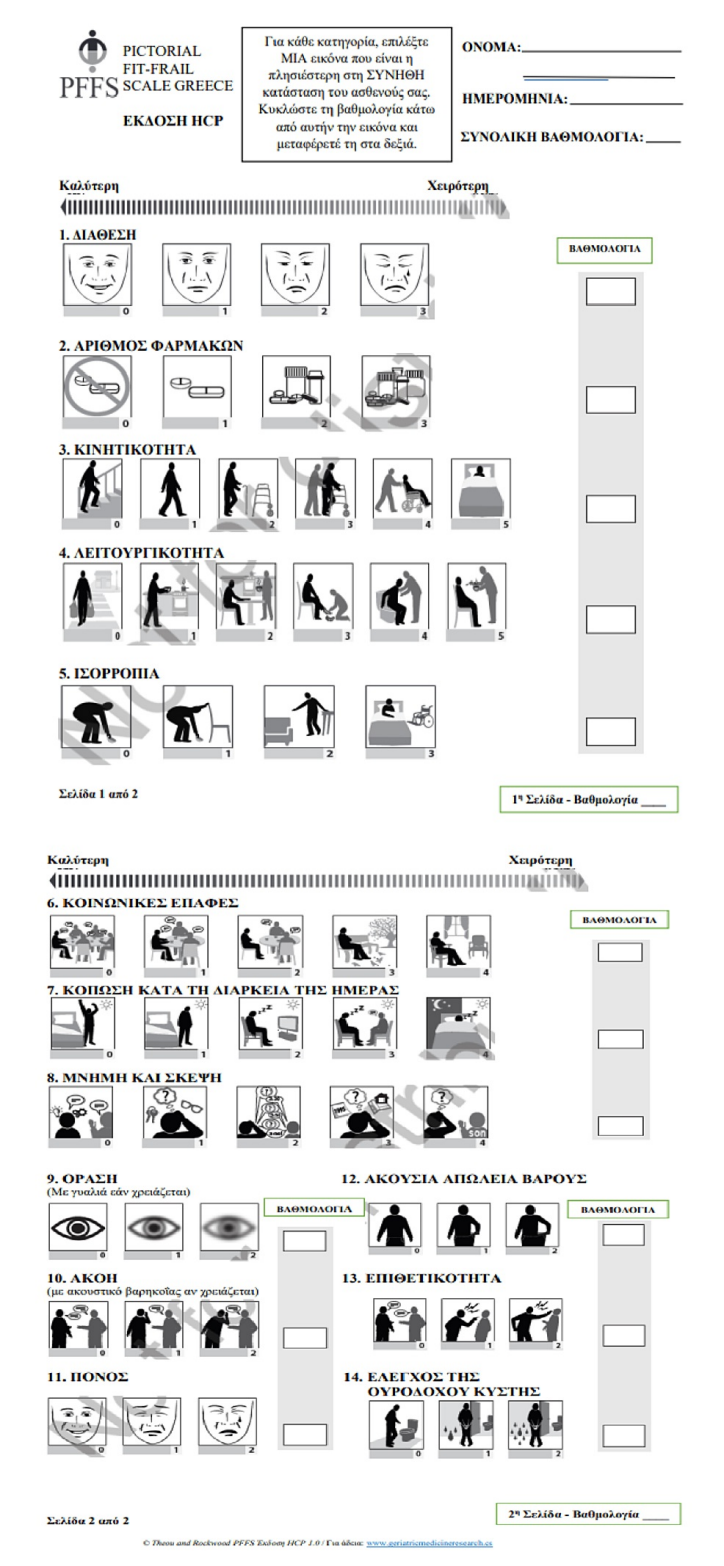
The final Greek version of the Pictorial Fit-Frail Scale (PFFS)

After getting the approval of the initial creators, two interpretations (independent and blinded) of the PFFS from English to Greek were carried out by two bilingual interpreters (one translation agency and a medical doctor with excellent certified knowledge of the English language). The two interpretations were compared, and an agreed choice of a suitable translation was made by the authors. At that point, the Greek version of PFFS was translated back into English by a professional translator and a native English speaker of Greek roots. Both re-translators were blinded to the first adaptation of the PFFS. We compared the two back-translated forms with the initial, and the contrasts were settled genially between the creators to improve the Greek interpretation. The Greek adaptation was at that point assessed by five physicians whose native language was Greek, and their comments were used to arrive at the final Greek adaptation (Figure 1).

Sample, setting, and data collection

A prospective pilot study was conducted on patients over the age of 65 years, who were subsequently hospitalized in the 2nd Department of Internal Medicine of the General and Oncology Hospital of Kifissia "Agioi Anargyroi" from August to November 2021. Age, sex, education level, marital status, medical history (comorbidities), drug use (number and type), and cause of hospitalization were all noted upon the patient's admission. Additionally, dependence on daily living activities was measured by the Barthel Index (BI), cognitive status was measured by the Global Deterioration Scale (GDS), and frailty was measured by using both PFFS and CFS and were assessed on patients’ admission. BI and GDS are not available in the Greek language, while CFS has been translated and validated in Greek [20]. CFS, BI, PFFS, and GDS were estimated for patients' baseline condition when not affected by acute illness. Information on demographics, medical history, drug use, and functional status was obtained by asking either the patient or the caregiver when the patient was not able to communicate.

For the purpose of the study, after the initial frailty assessment, the PFFS was noted for each patient (PFFS1). To assess inter-rater reliability, a second PFFS assessment was done by a different assessor who was blinded to previous scores (PFFS2). At least two weeks after reviewing all of the patient’s medical records, the first investigator re-evaluated the PFFS to assess the test-retest reliability (PFFS3).

Tools

Pictorial Fit-Frail Scale

The PFFS scale uses visual images to represent a range of domains associated with frailty. It assesses people's health status in 14 health domains, including mobility, function, cognition, social support, affect, medications, incontinence, vision, hearing, balance, and aggression. Each domain includes three to six levels that represent the quality of a person's health status. Within each domain, the evaluator selects the image that best reflects the patient's health status. Each domain tested in the tool is assigned a score of 0, which represents little or no disability level, and increasing scores indicate worsening health conditions or a higher level of disability. Total PFFS scores are calculated by adding the scores from each domain together. The sum of the scores for the 14 domains ranges from 0 to 43, with higher scores indicating increased frailty. A frailty index (FI) for the PFFS is constructed by dividing the total PFFS score by 43, the maximum score for this scale [12].

Clinical Frailty Scale

The validated Greek [20], revised nine-scale CFS was used to assess the frailty status of older adults [22]. CFS is an assessment-based measure of frailty that generates a score ranging from 1 (very fit) to 9 (terminally ill), based on an elderly person's overall fitness or frailty level [22].

Charlson Comorbidity Index

Comorbidity was evaluated using the Charlson Comorbidity Index (CCI). The CCI is a measure that accounts for the majority of important medical comorbidities. It includes 17 comorbidity categories and age groups, and each is associated with a score (from 1 to 6). A global comorbidity score is calculated by adding the individual scores for each patient [23].

Barthel Index

The BI was used to assess patients' performance in activities of daily living. BI is an ordinal scale from 0 to 100 that evaluates functional independence in 10 areas of personal care and mobility. A higher score corresponds to a greater ability to function independently [24].

Global Deterioration Scale

A seven-point scale was used to evaluate cognition, which can be further broken down into three groups: no cognitive decline, mild cognitive decline, and severe to very severe cognitive decline [25]. Stages range from no cognitive decline (stage 1) to very severe cognitive decline - severe dementia (stage 7).

Ethical approval

The study protocol was approved by the Ethical and Scientific Committee of the General and Oncology Hospital of Kifissia “Agioi Anargyroi” (approval number: 13/10805-07/08/2021). Written informed consent was obtained from patients or their relatives.

Validity and reliability of the Greek version of PFFS

By looking at potential correlations between sociodemographic and health-related traits and the degree of fitness or frailty as determined by the PFFS, the "known-group" construct validity of the PFFS was assessed. The existence of frailty was specifically predicted to be linked to older age, the presence of mobility and swallowing issues, social withdrawal, falls in the previous months, and a higher degree of cognitive impairment, comorbidity, and dependency in activities of daily living. The relationship between PFFS and CFS was investigated to assess criterion concurrent validity.

PFFS1 and PFFS2 scores were used to assess inter-rater reliability, and PFFS1 and PFFS3 scores were used to assess test-retest reliability, respectively.

Statistical analyses

IBM SPSS Statistics (version 22; IBM Corp., Armonk, NY) was used for all analyses. The distribution of the continuous variables was assessed using the Kolmogorov-Smirnov test. The interquartile range (IQR) and median are used to express the continuous variables CCI, CFS, and the number of medicines, which have non-Gaussian distributions. Age of the patients and PFFS are expressed as means with a standard deviation (SD) of one and were normally distributed. Percentages are used to express categorical variables. The degree to which the PFFS distinguishes between subgroups of the study sample that differ in age, CCI, mobility, balance, sociability, swallowing capacity, and amount of cognitive impairment was tested using a known group comparison to evaluate construct validity. For comparisons, the test for trends was applied. The results were deemed statistically significant if the p-value was ≤0.05. The criterion concurrent validity was assessed using Pearson's correlation coefficient by evaluating the extent to which PFFS is related to CFS. The inter-rater and test-retest reliability of the PFFS were evaluated using the intraclass correlation coefficient with 95% confidence intervals (CIs).

## Results

During the research period, 161 older adults were admitted to the medical ward through the emergency department. Four patients were excluded due to non-consent to participate (three women and one man). The age of the patients was 81.48 ± 8.62 years (mean ± SD). Among the study participants, 74 were women (47.1%), and 83 were men (52.9%). According to the CFS ratings, 94 patients (59.9%) were categorized as frail. Patients’ characteristics are presented in Table 1.

**Table 1:** Patients’ characteristics IQR: interquartile range; SD: standard deviation; CCI: Charlson Comorbidity Index; GDS: Global Deterioration Scale; CFS: Clinical Frailty Scale; PFFS: Pictorial Fit-Frail Scale.

	n = 157
Gender	
Male	83 (52.9%)
Age (±SD)	81.48 ± 8.62
CCI (median-IQR)	5.00 (4.00-7.00)
Number of medications (median-IQR)	6.00 (3.00-8.00)
PFFS (±SD)	14.69 ± 10.61
Marital status	
Married	75 (47.8%)
Unmarried	4 (2.5%)
Divorced	6 (3.8%)
Widowed	72 (45.9%)
Educational status	
Primary	95 (60.5%)
Secondary	29 (18.5%)
Technological education institution	20 (12.7%)
University	13 (8.3%)
Living alone	
Yes	27 (17.2%)
No	130 (82.8%)
Barthel Index (BI) groups	
No dependency (BI ≥ 95)	56 (35.7%)
Mild-moderate dependency (BI = 90–65)	51 (32.5%)
Moderate-severe dependency (BI = 60–25)	30 (19.1%)
Absolute dependency (BI ≤ 20)	20 (12.7%)
Degree of cognitive impairment	
No cognitive impairment	100 (63.7%)
Mild-moderate cognitive impairment (equivalent to GDS ≤ 5)	51 (32.5%)
Severe-very severe cognitive impairment (equivalent to GDS ≥ 6)	6 (3.8%)
CFS groups	
Frail	94 (59.9%)
Non-frail	63 (40 + E27:M34.1%)

Known-groups comparison revealed that PFFS distinguished well between subgroups of older adults who differed in age, dependency in activities of daily living, comorbidity, degree of cognitive impairment, mobility, balance, sociability, and swallowing ability. As hypothesized, the oldest adults were more dependent on activities of daily living and those with impaired cognitive status, mobility, balance, and swallowing, and those who were socially withdrawn had higher PFFS scores. The differences in PFFS scores across the subgroups of older patients were statistically significant and confirmed the expected relationships, supporting the construct validity of the instrument (Table 2).

**Table 2:** PFFS scores across subgroups of the elderly based on socio-demographic and health-related features * Derived from the test for trends. PFFS: Pictorial Fit-Frail Scale.

Socio-demographic and health-related features	n	PFFS score	Statistical significance*
		Μ ± SD	
		Age groups (years old)				
65-79	57	9.39 ± 8.50	
≥80	100	17.72 ± 10.53	p ≤ 0.001
Charlson Comorbidity Index groups			
2-3	17	4.82 ± 4.85	
4-5	64	14.30 ± 11.30	p ≤ 0.001
6-7	43	16.44 ± 9.30	
≥8	33	18.27 ± 10.13	
Barthel Index (BI) groups			
No dependency (BI ≥ 95)	56	5.04 ± 3.39	
Mild-moderate dependency (BI = 90–65)	51	13.20 ± 6.21	
Moderate-severe dependency (BI = 60–25)	30	22.70 ± 4.17	p ≤ 0.001
Absolute dependency (BI ≤ 20)	20	33.55 ± 2.54	
Aid use			
None	79	7.87 ± 6.49	
Stick	36	14.64 ± 6.07	p ≤ 0.001
Frame	17	20.53 ± 6.12	
Chair or bedridden	25	32.36 ± 3.66	
Falls in previous months			
No	125	13.78 ± 10.75	p = 0.033
Yes	32	18.25 ± 9.37	
Socially engaged			
Frequent	39	6.97 ± 5.92	
Occasional	69	11.83 ± 7.98	p ≤ 0.001
Not	49	24.88 ± 9.04	
Swallowing problems			
No	141	13.25 ± 9.73	p ≤ 0.001
Yes	16	27.44 ± 9.73	
Degree of cognitive impairment			
No cognitive impairment	100	8.88 ± 6.67	
Mild-moderate cognitive impairment	51	23.96 ± 8.07	p ≤ 0.001
Severe-very severe cognitive impairment	6	32.83 ± 5.81	

**Figure 2:**
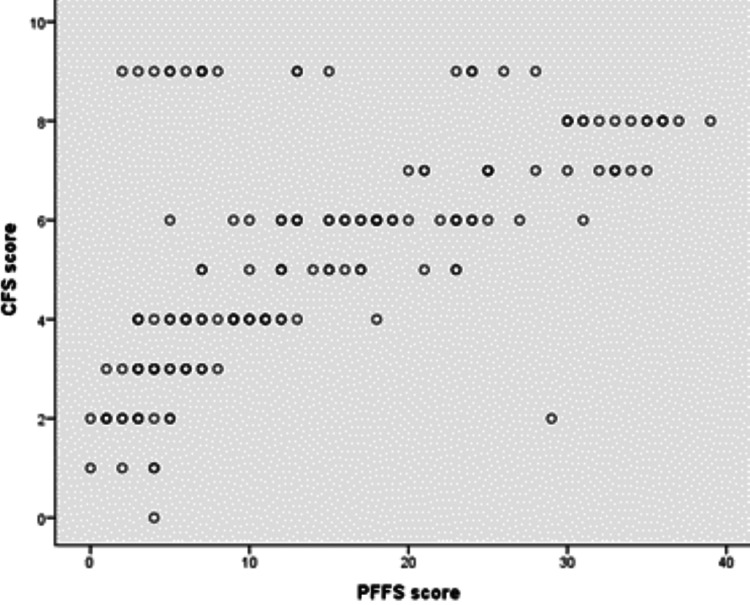
Scatter plot showing the relationship between CFS and PFFS scores. Pearson’s r = 0.625 CFS: Clinical Frailty Scale; PFFS: Pictorial Fit-Frail Scale.

Pearson's correlation coefficient (r) was applied to measure the association between CFS and PFFS. There was a moderate positive correlation among them, which was statistically significant (rs = 0.625, p ≤ 0.001), supporting the criterion concurrent validity of the instrument (Figure 2).

**Figure 3:**
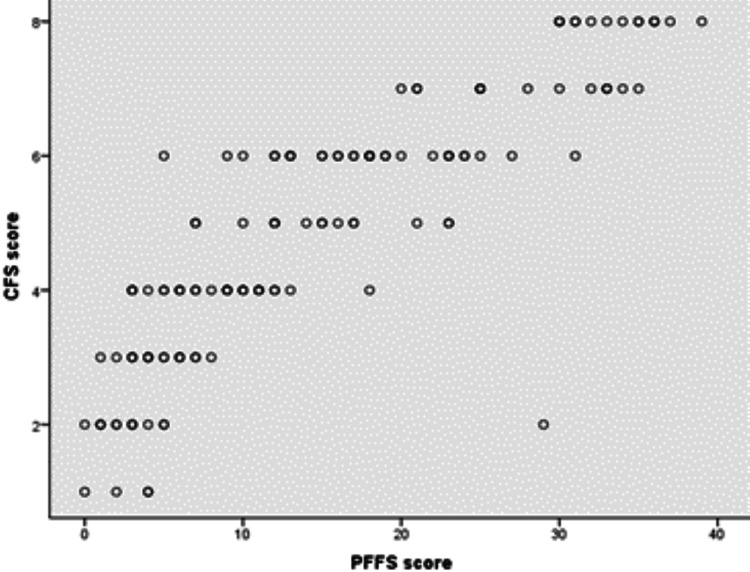
Scatter plot showing the relationship between CFS (excluding CFS = 9) and PFFS scores. Pearson’s r = 0.870 CFS: Clinical Frailty Scale; PFFS: Pictorial Fit-Frail Scale.

However, CFS includes category 9, which “applies to people with a life expectancy < six months, who are not otherwise living with severe frailty.” In PFFS, no such category exists. When we applied Pearson’s correlation coefficient (r) excluding persons categorized in category 9 in CFS, there was a strong positive correlation between CFS and PFFS, which was statistically significant (rs = 0.870, p ≤ 0.001) (Figure 3).

The intraclass correlation was good for inter-rater reliability, being 0.951 (95%CI: 0.934-0.964, p ≤ 0.001), and also for test-retest reliability, being 0.948 (95% CI: 0.930-0.962, p ≤ 0.001).

## Discussion

The aim of this study was to translate the PFFS into Greek and investigate its validity and reliability for the evaluation of frailty in elderly patients with a quick and easy-to-use instrument that is sensitive to varying levels of communication ability.

The study findings showed that the PFFS had a strong positive correlation with the CFS, supporting its criterion concurrent validity. Moreover, the ability of PFFS to distinguish between groups of older adults that differ in age, comorbidity, mobility, balance, dependency in activities of daily living, cognition, swallowing ability, and sociability provides evidence for its construct validity. Finally, the Greek version of PFFS exhibited excellent inter-rater and test-retest reliability.

Regarding the results for the test-retest reliability of our study, in the study by McGarrigle et al., the intraclass correlation coefficient was 0.88 for nurses, a result almost similar to ours [15]. Besides, test-retest reliability has been proven to be high when visual scales were implemented [26,27]. So, it is not surprising that in our study, the inter-rater reliability was also high. McGarrigle et al. [15] found weaker inter-rater reliability between nurses and doctors (intraclass correlation coefficient = 0.75), but in our study, frailty assessment was conducted from physicians only. The differences may represent training differences between these two professions.

Regarding the concurrent validity of PFFS, our study's result is in line with other studies that demonstrated its comparable diagnostic accuracy with other tools [13,14]. The differences in correlations between CFS and PFFS when we include or did not include category 9 of CFS was because practically, frailty and functional dependency progressed gradually until item 8. Category 9, by definition, applies to people with short life expectancy "who are not otherwise living with severe frailty," so, the physical limitation is not apparent [22]. So, when we included category 9 of CFS in correlation analysis, which includes patients that may be non-frail (and PFFS rate them as non-frail), the correlation became weaker.

Regarding the construct validity of PFFS, we ascertained differences in PFFS ratings in patients who differed in several domains that are commonly used for frailty assessment [28]. Most of them were proposed as essential components for the ideal frailty measures in the systematic review of de Vries et al. [29].

We are aware of our study limitations. First, our study sample is derived from the department of one hospital; thus, the study sample characteristics may be different from a sample derived from other healthcare settings or community-dwelling older adults. Second, we did not examine the feasibility of the instrument in patients, caregivers, or other healthcare professionals to identify whether the Greek version of PFFS could be rated reliable by untrained raters.

## Conclusions

PFFS has some unique characteristics, as it can be easily scored and the essential components of frailty can be distinguished by its use. The results of our study demonstrated that the Greek adaptation of PFFS has adequate psychometric properties for measuring frailty in older Greek adults. Its availability in the Greek language is expected to contribute to the assessment of older persons’ frailty, even by non-geriatricians.
